# Heartburn in children and adolescents in the presence of functional dyspepsia and/or irritable bowel syndrome correlates with the presence of sleep disturbances, anxiety, and depression

**DOI:** 10.1097/MD.0000000000025426

**Published:** 2021-04-02

**Authors:** Jennifer M. Colombo, Amanda D. Deacy, Jennifer V. Schurman, Craig A. Friesen

**Affiliations:** a Division of Pediatric Gastroenterology, Children's Mercy Kansas City; b University of Missouri Kansas City School of Medicine, Kansas City, MO.

**Keywords:** functional dyspepsia, heartburn, irritable bowel syndrome, sleep disturbances

## Abstract

The aim of this study was to assess the relationship of heartburn in pediatric patients with functional dyspepsia (FD) and irritable bowel syndrome (IBS) with gastrointestinal symptoms, sleep disturbances, and psychologic distress.

The overlap in symptoms of FD, IBS, and gastroesophageal reflux disease (GERD) predicts greater symptom severity and decreased quality of life and presents opportunities for improved diagnostic classification and personalized therapeutics.A cross-sectional observational study of 260 pediatric patients with abdominal pain was conducted. Patients completed standardized questionnaires assessing clinical symptoms, sleep quality, and psychologic symptoms during routine clinical care. Questionnaire data were compared for patients reporting heartburn and not reporting heartburn using *χ*^2^ and *t* tests where appropriate.

Gastrointestinal symptoms were significantly more prevalent among patients with a positive report of heartburn (vs a negative report of heartburn): pain with eating (83% vs 67%, *P* = .007), bloating (63% vs 44%, *P* = .005), acid regurgitation (47% vs 24%, *P* ≤ .001), and chest pain (45% vs 20%, *P* ≤ .001). Likewise, initiating and maintaining sleep (*P* = .007), arousal/nightmares (*P* = .046), sleep-wake transition (*P* = .001), hyperhidrosis during sleep (*P* = .016), and anxiety (*P* = .001) and depression (*P* = .0018) were also significantly increased in patients who reported heartburn versus patients who did not report heartburn.

Patients with a positive report of heartburn, whether classified as having FD and/or IBS, had increased gastrointestinal symptoms, sleep disturbances, anxiety, and depression than patients with a negative report of heartburn. A better understanding of these associations may allow for personalized treatment for youth with abdominal pain and heartburn as a primary symptom.

## 1 Introduction

Functional gastrointestinal disorders associated with abdominal pain (FGID-AP) as defined by symptom-based Rome IV criteria are common disorders in children and adolescents.^[1]^ The two most frequent FGID-APs are functional dyspepsia (FD) and irritable bowel syndrome (IBS). FD is defined as the presence of epigastric pain or burning unrelated to stools, early satiety, or post-prandial fullness.^[2,3]^ IBS is defined by the presence of pain related to a change in the stool frequency, a change in the stool consistency, or by a change in pain with stools.^[2,3]^ Classification of the disorders by symptomatology is required because there is no identified underlying mechanism or trigger for the disorders to aid in the objective diagnosis.^[2]^ Although diagnostic criteria in the form of Rome IV exist, agreement in the interpretation of the symptoms remains problematic as the symptoms often overlap.^[4,5]^ What is known, however, is that these disorders are multifaceted and involve a complex interplay of biological, psychological, and social factors, and that there is no single effective treatment.^[1,6]^

The overlap of symptoms in patients with FGID-APs, while complex, may represent an opportunity for improved classification schemes and more individualized treatment options. Commonly identified and reported in the literature are the frequent overlap of FD, IBS, and gastroesophageal reflux disease (GERD) symptoms.^[1,5,7–11]^ Symptoms of GERD include heartburn and regurgitation; however, the symptom of heartburn may not always be related to acid exposure in the esophagus.^[12]^ GERD can be further classified based on symptoms using symptom-based Rome IV criteria and findings during endoscopic evaluation into non-erosive esophagitis (NERD), acid reflux hypersensitivity, and functional heartburn.^[13]^ The prevalence of Rome IV nonerosive phenotypes has been described in children and the most common phenotype identified is functional heartburn defined as normal esophageal acid exposures and negative symptom association.^[14]^

The overlap of FD, IBS, and GERD symptoms tends to predict worsening clinical symptoms and decreased quality of life in both pediatric and adult patients.^[7–11]^ Additionally, FD, IBS, and GERD symptoms are associated concurrently with other nongastrointestinal symptoms, including sleep disturbances, anxiety, and depression. These non-gastrointestinal symptoms can contribute to the maintenance, or even progression, of the disorders.^[4,9,15,16]^

Among the non-gastrointestinal correlates of FD, IBS, and GERD, sleep disturbances are especially common in pediatric and adult patients with chronic gastrointestinal symptoms.^[17,18]^ Altered brain–gut interactions can influence circadian rhythm and sleep regulation, thereby contributing to the strong associations identified between sleep disturbances and functional gastrointestinal disorders.^[18–24]^ Furthermore, the association of gastroesophageal reflux symptoms and sleep disturbances appears to be bidirectional;^[18,21]^ that is, symptoms of GERD (eg, heartburn) can disrupt sleep and disrupted sleep, in turn, can aggravate symptoms of GERD. Finally, sleep disturbances are common in patients with psychological disorders as sleep disturbance is a diagnostic feature of both anxiety and depressive disorders which adds to the complexity of the interactions between gastrointestinal symptoms, sleep disturbances, and psychological distress.^[19,20,23,25,26]^

Identifying and understanding characteristics of specific FGID-AP phenotypes may shed light on potential personalized therapeutic options.^[10]^ Given the relationship of GERD symptoms, such as heartburn, and the observed overlap between GERD, FD, and IBS, the specific symptom of heartburn appears to be a reasonable place to begin examining symptom phenotypes that cut across FGID-AP classifications. Furthermore, understanding the concurrent associations with sleep, anxiety, and depression, could provide additional meaningful points of differentiation among phenotypic profiles to drive biopsychosocial treatment choices. Thus, the primary aims of this study were to assess the rates of co-occurring heartburn (as a symptom of GERD) in pediatric patients with FGID-AP, namely FD and IBS, and to assess the association of heartburn presence with sleep disturbances, psychologic symptoms, esophagitis, and specific gastrointestinal symptoms. We hypothesized that co-occurrence of heartburn and FGID-APs, based on adult studies, would be frequent. Furthermore, we hypothesized that youth presenting with heartburn would have higher rates of problems with sleep, anxiety, and depressed mood relative to youth without heartburn. Given the previous suggestion that the symptom of heartburn may not always be related to acid exposure in the esophagus, we also hypothesized that esophagitis would not be universal in our sample of patients reporting heartburn. Finally, associations with specific GI symptoms were explored to help elucidate possible phenotypes of GERD.

## 2 Methods

The present study procedures were approved by the institutional review board at Children's Mercy Kansas City.

We conducted a cross-sectional retrospective chart review of a convenience sample of 260 consecutive patients, 8 to 17 years of age, with a primary complaint of abdominal pain occurring at least once weekly for a minimum of 8 weeks between May 1, 2013 and August 1, 2016. Inclusion criteria specified patients 8 to 17 years of age who were seen for initial evaluation of a complaint of chronic (at least 8 weeks) of abdominal pain in a single interdisciplinary Abdominal Pain Clinic at a medium-sized, Midwestern pediatric academic medical center. Patients were excluded if they did not have a diagnosis of a Functional Gastrointestinal Disorder associated with pain (FGID-AP) or were found to have a primary diagnosis of another organic gastrointestinal condition (eg, IBD < celiac) upon workup preceding or following the initial evaluation. Seventeen patients were excluded due to not meeting criteria for functional GI disorders (ie, diagnosed with an organic GI disease). Patients were diagnosed with an FGID-AP, using Rome IV criteria, by a single board-certified pediatric gastroenterologist at the time of their initial evaluation visit.

As part of their routine, initial clinical evaluation, all patients completed a standardized medical history form and standardized questionnaires. The medical history form included questions regarding the abdominal pain complaint and associated gastrointestinal and nongastrointestinal symptoms. Sleep quantity and quality were assessed using the parent-reported *Sleep Disturbances Scale for Children* (SDSC).^[27]^ Emotional symptoms were assessed using the Anxiety and Depression subscales of the *Behavior Assessment Systems for Children, 3*^*rd*^* Edition* (BASC-3), which was administered to children (ages 8–11), adolescents (ages 12–18), and their parents.^[28]^

Additionally, all patients with FD and FD overlap with IBS (n = 240) had undergone upper endoscopy with biopsies after failing to respond to acid reduction therapy. At least 2 biopsies were obtained from the distal one-third of the esophagus, gastric antrum, and duodenum. All patients were negative for erosive esophagitis, nodularity, erosions, and *Helicobacter pylori*. The pathology reports were reviewed for the presence of microscopic esophagitis as defined by increased thickness of the basal cell layer, increased length of the papillae, and intraepithelial inflammation including eosinophils (0-14/hpf).

### 2.1 Statistical analysis

Statistical analyses were performed using SPSS version 23 (SPSS Inc., Chicago, IL). Frequency counts were obtained for each assessed gastrointestinal and nongastrointestinal symptom and resulting FGID-AP diagnoses. More detailed frequency comparisons, using *χ*^2^, were conducted to determine the frequency of heartburn in patients with FD alone, IBS alone, and both FD and IBS, as well as in males versus females, younger versus adolescent patients (ie, age ≤12 years and age >12 years), and patients with and without histologic esophagitis. The frequencies of specific gastrointestinal symptoms and nongastrointestinal symptoms also were compared in patients who reported heartburn and those who did not report heartburn using *χ*^2^ analyses.

Independent samples *t* tests were used to compare mean SDSC and anxiety and depression subscale scores in patients who reported heartburn and those who did not. A *P* value of <.05 was considered statistically significant for all analyses.

## 3 Results

In general, 39% of patients met Rome IV criteria for FD only and 8% met criteria for IBS only. Fifty three percent of patients fulfilled criteria for both FD and IBS. Overall, heartburn was reported by 38% of patients. Heartburn was reported by 27% of patients with FD alone, 35% of patients with IBS alone, and 46% of patients fulfilling criteria for both FD and IBS (*P* = .018). There were no differences in sex and age between the groups of patients fulfilling criteria for FD, IBS, or both FD and IBS. Histologic esophagitis was identified in 26% of the patients who reported heartburn and 17% of the patients who did not report heartburn (*P* = .124).

Pain with eating, bloating, acid regurgitation, and chest pain were significantly more common in patients who reported heartburn compared to those who did not report heartburn (Table 1). Initiating and maintaining sleep, arousal/nightmares, sleep-wake transition, and hyperhidrosis during sleep were significantly increased in patients who reported heartburn versus patients who did not report heartburn (Table 2). All psychological functioning scores (both self-report and parental report) were significantly increased in patients who reported heartburn compared to patients who did not report heartburn (Table 3).

**Table 1:** Percentage of GI in patients diagnosed with FGID-AP and reporting heartburn vs not reporting heartburn.

Symptom	Heartburn (%)	No Heartburn (%)	*P*
Epigastric pain	49	39	.127
Pain at night	60	54	.368
Pain with eating	83	67	.007
Early satiety	74	67	.219
Bloating	63	44	.005
Burping	16	12	.327
Acid regurgitation	47	24	<.001
Chest pain	45	20	<.001

FGID-AP = functional gastrointestinal disorders associated with abdominal pain.

**Table 2:** Sleep scores in patients with diagnosed with diagnosed with FGID-AP and reporting heartburn vs not reporting heartburn.

Parameter	Heartburn	No Heartburn	*P*	Cohen d
DIMS	17.05 ± 5.68	15.04 ± 4.89	.007	0.379
SBD	3.92 ± 1.53	3.68 ± 1.36	.234	0.166
DA	3.72 ± 1.27	3.39 ± 0.913	.046	0.299
SWTD	10.54 ± 3.60	8.49 ± 2.61	<.001	0.653
DOES	9.92 ± 4.17	9.09 ± 3.41	.111	0.218
SHY	3.22 ± 2.39	2.50 ± 1.30	.016	0.372

DA = arousal/nightmares, DIMS = initiating and maintain ning sleep, DOES = excessive somnolence, FGID-AP = functional gastrointestinal disorders associated with abdominal pain, SBD = sleep breathing, SHY = sleep hyperhidrosis, SWTD = sleep/wake transition.

**Table 3:** Psychologic scores in patients diagnosed with diagnosed with FGID-AP and reporting heartburn vs not reporting heartburn.

Score	Heartburn	No Heartburn	*P*	Cohen d
Anxiety (self)	57.73 ± 11.46	51.81 ± 10.57	<.001	0.537
Depression (self)	51.2 ± 10.12	48.19 ± 9.94	.018	0.315
Anxiety (parent)	60.4 ± 12.06	56.23 ± 12.71	.013	0.337
Depression (parent)	58.51 ± 11.22	55.03 ± 12.64	.033	0.291

FGID-AP = functional gastrointestinal disorders associated with abdominal pain.

## 4 Discussion

The symptom of heartburn was positively reported by 38% of the patients in this study. This subgroup reported significantly more abdominal pain with eating, bloating, acid regurgitation, and chest pain than those without heartburn. It is not surprising that these specific gastrointestinal symptoms are significantly increased in patients with FGID-APs who reported heartburn as these symptoms are common overlap symptoms of gastroesophageal reflux disease. This is consistent with the findings of Hsu et al^[9]^ who studied adult patients with GERD and FD versus GERD alone. The patients with GERD and FD had more severe GERD symptoms when compared with adult patient with GERD alone.

In the present study, there was no difference in age, sex, or histologic finding of esophagitis for the patients reporting heartburn which is consistent with previous studies. Quitadamo et al^[29]^ demonstrated no correlation between reflux symptoms, symptom severity, and the histologic grade of esophagitis in children and adolescents even when focusing only on adolescents with heartburn or chest pain. Mahoney et al^[14]^ identified functional heartburn (normal esophageal acid exposure and negative symptom index) as the most common Rome IV non-erosive esophageal phenotype in children and microscopic esophagitis and response to proton pump inhibitor medication did not predict the GERD phenotype. This suggests that treatment with proton pump inhibitor alone may not be sufficient for patients who complain of heartburn symptoms. Although the symptom of heartburn has associations, it is not clear whether the symptom is true acid-related heartburn or functional heartburn.

Sleep disturbances, and anxiety and depression are associated with functional abdominal pain^[18]^ and these associations are particularly salient in this study of patients reporting heartburn versus patients not reporting heartburn regardless of specific FGID-AP diagnoses. The current subgroup of patients with FGID-APs and heartburn reported more disrupted sleep (ie, initiating and maintaining sleep, night-time arousals/nightmares, sleep wake transition, and hyperhidrosis during sleep) than those who did not report heartburn. Previous adult studies have also demonstrated this trend in patients diagnosed with a functional gastrointestinal disorder (FD and IBS) who reported heartburn. Iwakura et al^[23]^ described sleep disturbances in approximately half of their patients with GERD according to the Pittsburg Sleep Quality Index.

Lei et al^[18]^ compared a sample of adults with and without known sleep difficulties/disturbances. Results indicated that those with identified sleep problems had a higher prevalence of FD, IBS, and more severe symptoms of GERD, as well as higher scores on measures of anxiety and depression. Further, in the study by Lindam et al,^[21]^ sleep disturbances and reflux symptoms were bidirectionally associated. There was a significantly increased risk of developing reflux symptoms among individuals with sleep disturbances and a moderately increased risk of developing sleep disturbances among individuals with reflux symptoms. The mechanism for this is not completely understood; however, night-time awakenings due to reflux symptoms lead to poorer sleep quality. Monzon et al^[30]^ compared adolescents with and without FGIDs and noted that adolescents with FGIDs obtained significantly less sleep and that when adolescents have lower emotional distress, they achieve longer sleep durations. There is also some preliminary evidence that better sleep quality predicts overall improvement, and speed of improvement, of gastrointestinal complaints in children.^[31]^

The subgroup of our patients reporting heartburn also had significantly greater anxiety and depression scores relative to their FGID-AP counterparts without heartburn. This finding is also similar to adult studies. Bilgi et al^[32]^ compared adult patients with GERD versus healthy controls and noted a significant association between all GERD subtypes and depressive disorders. A large cross-sectional study by Choi et al^[15]^ also revealed significantly higher anxiety and depression levels in patients with GERD. Additionally, Kimura et al^[22]^ demonstrated that 47% of patients with GERD were partial responders to proton pump inhibitor treatment and these patients had significantly more sleep problems, anxiety, depression, and general mental health concerns in comparison to complete responders. A study by Li et al^[33]^ evaluated adult patients with nonerosive reflux disease randomized to receive omeprazole plus domperidone only, cognitive behavioral therapy (CBT), or omeprazole plus domperidone and CBT. Patients in the medication plus CBT group showed the best overall improvement suggesting co-existence of mental health concerns and heartburn and reflux symptoms. Identifying co-morbid mental health concerns, even when they do not rise to the level of diagnosable disorders in children with functional abdominal pain may help guide a comprehensive approach to treating these disorders.

An improved understanding of the associations among gastroesophageal reflux disease symptoms, namely heartburn, sleep disturbances, and anxiety and depression, from biological, sensory, and psychological perspectives, could reveal important treatment targets for pediatric patients with functional abdominal pain. It is necessary to further understand this connection because treatment of heartburn alone with acid suppressing agents does not resolve the remaining symptoms.^[8,14]^ The symptom of heartburn has its own unique associations with other GI symptoms, sleep disturbances, anxiety, and depression across youth with FD, IBS, and FD/IBS, and with this understanding, there may be greater implications for treatment. Children and adolescents with FGID-APs should be screened for symptoms of heartburn, sleep disturbances, and psychological symptoms as part of their clinical evaluation in order to devise a more comprehensive approach to treatment.

The resounding strength of the present study is the standardized collection of medical history, including gastrointestinal symptom data, along with psychosocial data in a large sample of children and adolescents with FGID-AP. Limitations of the study include its retrospective and cross-sectional nature which allows only for descriptive associations to be established. Future studies are needed to explore the directional nature of the relationship between functional gastrointestinal disorders, specific gastrointestinal complaints (including true acid-related heartburn and functional heartburn), sleep disturbances, and mental health issues. Furthermore, there is a body of work pointing toward early life experiences (eg, early childhood maltreatment) as “sensitizing” events that impact nerve pathways to leave youth more susceptible to further psychological and physical morbidity.^[34,35]^ Although early life experiences have been shown to be associated with the development of later GI symptoms, including FGID-APs, assessment of these was not part of the available standardized clinical history. Studies examining the impact of heartburn treatment on other GI symptoms, sleep, anxiety, and depression also may help better explicate the pathophysiology and directionality of relationships to support more targeted care. Similarly, it would be helpful to note whether treatment of other concurrent GI and non-GI symptoms also improves heartburn. We would encourage future treatment studies to include measures of each variable to understand the downstream impacts of specific treatments on various symptom phenotypes.

In conclusion, in youth already diagnosed with FGID-AP, having a positive report of heartburn is associated with a greater likelihood of experiencing concurrent specific gastrointestinal symptoms, sleep disturbances, anxiety, and depression, compared to those without heartburn. This suggests that, in clinical practice, we should be routinely assessing for heartburn in our pediatric FGID-AP patients. The index of suspicion for further sleep and emotional problems should be higher when heartburn is present and consultation with a pediatric psychologist and/or sleep medicine expert should be considered to investigate these issues more fully. The presence of heartburn, in effect, may signal the need for a more personalized approach to management and treatment even within the group of children with functional gastrointestinal disorders. More broadly, this indicates that current existing classification systems may be inadequate to fully capture the nuance and needs of various FGID-AP phenotypes. Although we have started with the symptom of heartburn, it remains possible that another specific GI symptom could similarly be a marker for a meaningful FGID-AP phenotype. Ultimately, the existing Rome IV classification system provides a starting point for assessment but identifying the additional variables that signal a need to shift treatment in one direction or another from the “typical” has the potential to lead to more personalized and effective treatment.

## Author contributions

J.C., A.D., J.S., and C.F. participated in study design. C.F. completed the data analysis. A.D. and J.S. had oversight of the statistical analysis. J.C. and A.D. wrote the main draft of the manuscript. J.C., A.D., J.S., and C.F. critically reviewed and revised the final manuscript.

**Conceptualization:** Jennifer M. Colombo, Amanda D. Deacy, Jennifer V. Schurman, Craig A. Friesen.

**Data curation:** Jennifer M. Colombo, Amanda D. Deacy, Jennifer V. Schurman, Craig A. Friesen.

**Formal analysis:** Craig A. Friesen.

**Investigation:** Jennifer M. Colombo, Amanda D. Deacy.

**Methodology:** Jennifer M. Colombo, Amanda D. Deacy, Jennifer V. Schurman, Craig A. Friesen.

**Supervision:** Craig A. Friesen.

**Validation:** Jennifer M. Colombo, Amanda D. Deacy, Jennifer V. Schurman, Craig A. Friesen.

**Visualization:** Jennifer M. Colombo, Amanda D. Deacy, Jennifer V. Schurman, Craig A. Friesen.

**Writing – original draft:** Jennifer M. Colombo, Amanda D. Deacy.

**Writing – review & editing:** Jennifer M. Colombo, Amanda D. Deacy, Jennifer V. Schurman, Craig A. Friesen.
